# Potential of epigenetic therapies in non-cancerous conditions

**DOI:** 10.3389/fgene.2014.00438

**Published:** 2014-12-19

**Authors:** Theresa Mau, Raymond Yung

**Affiliations:** ^1^Division of Geriatric and Palliative Medicine, Department of Internal Medicine, University of MichiganAnn Arbor, MI, USA; ^2^Department of Veterans Affairs Ann Arbor Health System, Geriatric Research, Education and Clinical Care CenterAnn Arbor, MI, USA

**Keywords:** epigenetics, autoimmunity, therapies, diabetes mellitus, obesity, diet

## Abstract

There has been an explosion of knowledge in the epigenetics field in the past 20 years. The first epigenetic therapies have arrived in the clinic for cancer treatments. In contrast, much of the promise of epigenetic therapies for non-cancerous conditions remains in the laboratories. The current review will focus on the recent progress that has been made in understanding the pathogenic role of epigenetics in immune and inflammatory conditions, and how the knowledge may provide much needed new therapeutic targets for many autoimmune diseases. Dietary factors are increasingly recognized as potential modifiers of epigenetic marks that can influence health and diseases across generations. The current epigenomics revolution will almost certainly complement the explosion of personal genetics medicine to help guide treatment decisions and disease risk stratification.

## INTRODUCTION

Epigenetics is traditionally defined as heritable gene expression modifications that cannot be attributed to changes in the primary deoxyribonucleic acid (DNA) sequence. The epigenetic machinery is particularly important in organismal development where stable and distinct cellular functions must be established from an identical genotype. As such, efforts have been placed into decodifying the epigenetic language that is believed to regulate gene expression. In a growing amount of complex diseases, researchers are identifying epigenetic dysregulation that can be associated to their pathogenesis, and although studies can vaguely shed light on whether epigenetics as a product or an effect of the disease, evidence indicates that epigenetics play a substantial role in the susceptibility of an individual to these diseases.

Deoxyribonucleic acid methylation and histone modifications are considered to be the two major epigenetic mechanisms, but other epigenetic mechanisms include alterations in ribosomal DNA (rDNA) and microRNA (miRNA). Both non-covalent and covalent modifications can affect chromatin structure and cause remodeling to it in such a way that it influences heritable gene expression. DNA methylation leads to gene silencing, whereas posttranslational modifications of histone proteins can lead to either induction or repression of gene activity. These epigenetic mechanisms are believed to be crucial for reading environmental stimuli and creating long-lasting changes that could be passed onto future generations (Manolio et al., 2009). Genome-wide association studies (GWAS) are moving toward including epigenetic analysis (epigenomics) alongside the analysis of single nucleotide polymorphisms (SNPs) in individuals to build a more complete hereditary profile for complex diseases.

Epigenetic pathways have long been recognized as a major player in oncogenesis. The influence of the epigenome have been associated to many key aspects of cancer, such as malignant self-renewal, differentiation blockade, evasion of cell death, and tissue invasiveness (Dawson and Kouzarides, 2012). As such, the development of epigenetic drugs to counter these aspects of cancer has been one of the focuses of cancer research. For example, because cancerous conditions can stem from a complexity of changes in epigenetic regulators, inhibitors of DNA methyltransferases and histone deacetylases (HDACs) have been approved by the US Food and Drug Administration for therapeutic purposes. However, now that a tremendous amount of evidence is mounting to show that many other diseases are affected by the epigenome, drug repositioning efforts have been launched to explore epigenetic therapies for non-oncology conditions (Best and Carey, 2010). This review aims to focus on the epigenetics of immune diseases and to highlight the therapeutic potential of epigenetic interventions.

## DNA METHYLATION

**FIGURE 1:**
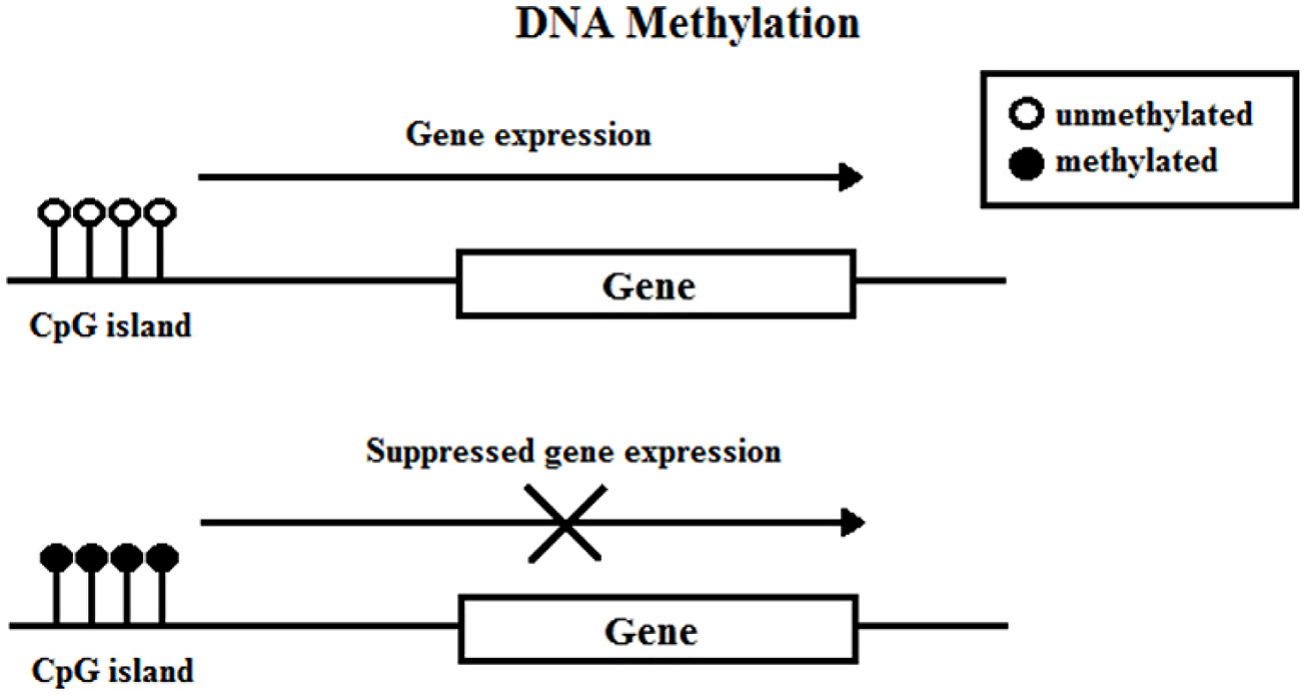
**DNA methylation**.

Deoxyribonucleic acid methylation involves the covalent attachment of a methyl group onto the C5 position of a cytosine residue on CpG (cytosine-phosphate-guanine) islands (**Figure 1**). A group of enzymes, DNA methyltransferases (DNMTs), catalyze the addition of the methyl group to the DNA; the DNMT family includes DNMT1, DNMT3a, DNMT3b, and DNMT3l. DNA methylation typically occurs during DNA replication to maintain methylation patterns down cell lineages via hemimethylated DNA, but during replication and embryogenesis, it is also possible for *de novo* methylation of DNA to occur. The family of methyl-CpG-binding proteins consists of six members thus far, MBD1–MBD4, Kaiso, and methyl-CpG binding protein 2 (MeCP2) (Fan and Hutnick, 2005; Klose and Bird, 2006).

Feng and Fan (2009) concisely discussed four current models in which DNA methylation can mediate gene silencing. DNA methylation can prevent the transcriptional activator binding to the target DNA sequence which directly impedes transactivation (Watt and Molloy, 1988). Next, the DNMT protein might be physically linked to HDAC and histone methylase (HMT) proteins, allowing the coupling of enzymatic activities (Fuks et al., 2000). Third, DNA methylation within the gene body exerts a repressive effect on transcriptional elongation—it can occur in the promoter or downstream (Lorincz et al., 2004). Lastly, methyl-CpG-binding proteins have been shown to directly recognize methylated DNA and recruit transcriptional repressors to silence and modify surrounding chromatin (Nan et al., 1997, 1998).

It is important to recognize that the epigenetics field is rapidly expanding and changes in other epigenetic processes, such as DNA hydroxymethylation (implicated in DNA demethylation), may be important in disease pathogenesis and are therefore potential therapeutic targets as well. Tet enzymes (Tet1/2/3) convert 5-methylcytosine (5mC) to 5-hydroxymethylcytosine (5hmC) and possibly 5-formylcytosine (5fC) and 5-carboxylcytosine (5caC) (Wu and Zhang, 2011; Huang et al., 2014). Tet3 has recently been implicated in a study to significantly contribute to hydroxylation of 5mC during development. The key finding in this study, however, was on Tet1 and Tet2; the study shows that Tet1/Tet2-deficient mice are viable and produces fertile offspring but accrued developmental defects attributed to a reduction in 5hmC and an increase in 5mC that was also accompanied by hypermethylation (Dawlaty et al., 2013). The epigenetic anomalies and disruption in somatic genomic reprogramming in these mice embryonic stem cells strongly suggest their role as regulators of DNA methylation and potential as therapeutic targets for autoimmune diseases.

## HISTONE MODIFICATIONS

Chromatin is described to form either euchromatin (DNA lightly wrapped around histone proteins that form nucleosomes and typically contains the most active genes) or heterochromatin (the tightly packed DNA). Enzymatic modifications to histones alter the structure of chromatin and influence the expression and repression of genes. The nucleosome is the basic repeat unit of chromatin which contains two copies of each histone (H2A, H2B, H3, and H4) that are wrapped by 146 base pairs of DNA (Li, 2002).

The posttranslational histone modifications include acetylation, methylation, phosphorylation, ubiquitination, ADP ribosylation, deimination, proline isomerization, and sumoylation. Of these, acetylation and methylation have received the most attention. These modifications determine the higher-order chromatin structure that can lead to both expression and repression of genes (Jenuwein and Allis, 2001). There is a “histone code” which is believed to be the combination of different histone modifications that define different epigenetic marks and these would then collectively generate the plasticity in gene expression among individuals.

**FIGURE 2:**
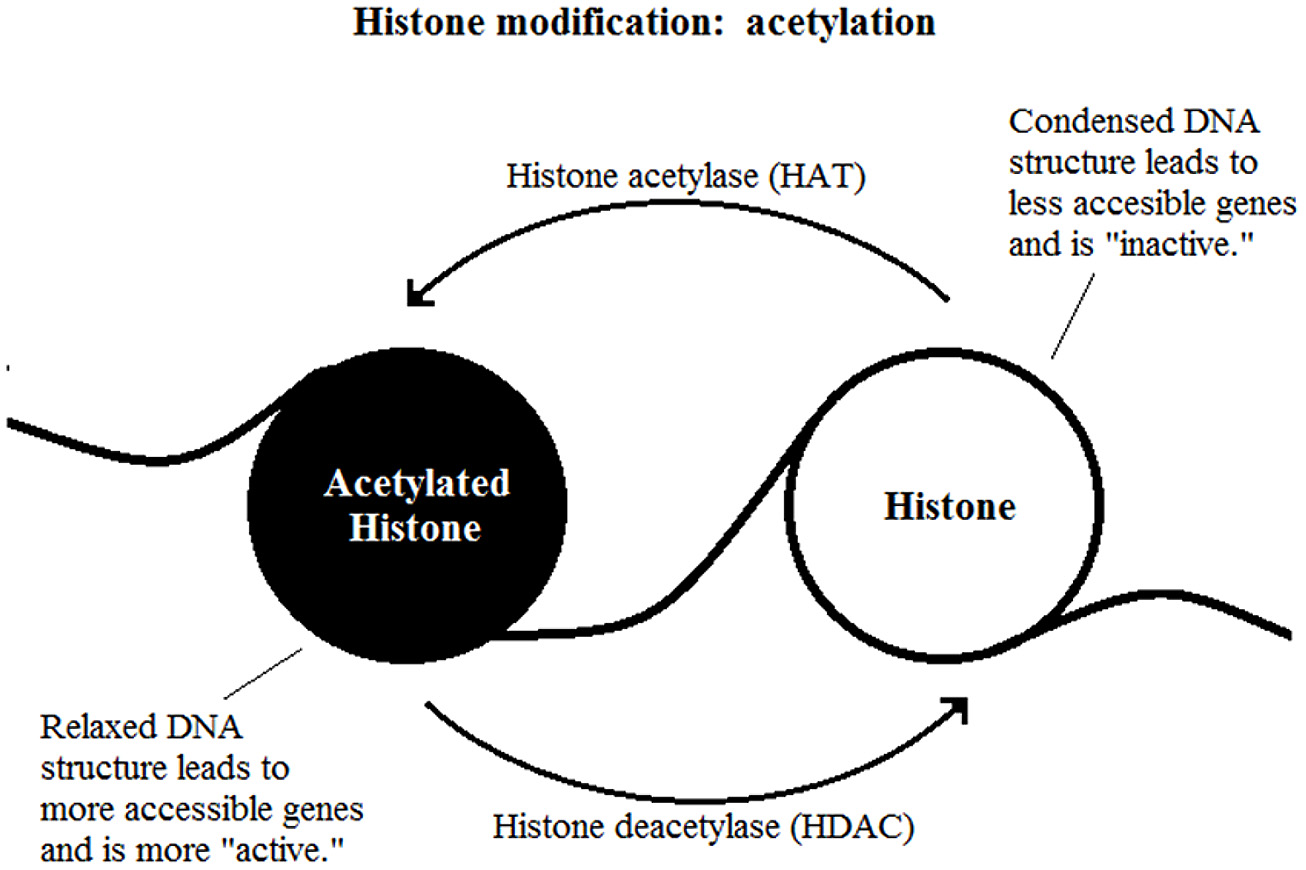
**Histone modification: acetylation**.

The most well-studied histone modification is the acetylation of lysine residues. It is a reversible process catalyzed by two enzymes, the histone acetylase (HAT) and HDAC (**Figure 2**). The presence of an acetyl group decreases interaction between negatively charged DNA and the positively charged histone tail which results in a less compact nucleosome, enables easier access for transcription factor complexes (Feng and Fan, 2009). Therefore, removal of the acetyl group by HDAC leads to gene transcription repression.

The effect of methylation of histones is based on how many methyl groups (mono-, di-, or tri-) are added onto the lysine residue. Tri-methylation on H3K4 (histone 3, lysine 4) is associated with transcriptional activation whereas the same methylation on H3K9 or H3K27 are highly indicative of transcriptional inhibition (Kouzarides, 2007). Methylation of histones is reversible through a process called histone demethylation (Shi et al., 2004; Klose et al., 2006).

## microRNA

Deoxyribonucleic acid methylation and histone modifications regulate short non-coding RNA called miRNAs that in turn regulate gene expression post-transcriptionally. However, a different subset of miRNAs behaves in the opposite way and can regulate the expression of epigenetic machines, such as the aforementioned DNMTs and HDACs, and polycomb group genes (Sato et al., 2011). These miRNAs are short in length, ranging between 19 and 25 nucleotides, and they are non-coding RNAs that participate in RNA interference (RNAi) machinery via binding to its target mRNA to decrease its translation (by guiding it to degradation or suppression) and thus making those transcripts less stable. RNA polymerase II (pol II) transcribes miRNA genes into primary miRNA transcripts that require further processing prior to its export from the nucleus (Lee et al., 2004).

In the past few years, the research interests in miRNAs and their role in regulation and participation of the epigenetic machinery have shown to be constructive for the deeper understanding of what the function of these short non-coding sequences encompass that influence autoimmunity. For example, miRNAs miR-23b, miR-30a have been shown to be downregulated and miR-146a and miR-214 upregulated in autoimmune disease samples such as human lupus, rheumatoid arthritis (RA), osteoarthritis (OA), and murine models of autoimmunity [experimental autoimmune encephalomyelitis (EAE), MRL/lpr, collagen-induced arthritis (CIA)] (Zhu et al., 2012b). Most interestingly, this study found that in human fibroblast-like synoviocytes, mouse primary kidney cells and astrocytes, miR-23b can be downregulated by IL-17. However, miR-23b was found to be a suppressor of IL-17-associated autoimmune inflammation (IL-17, TNF-α, IL-1β-induced NF-κB activation) through specific target binding of TGF-β-activated kinase 1/MAP3K7 binding protein 2 (TAB2) and TAB3 which is an inhibitor of NFκ-B subunit α (IKK-αa). Through these findings, it was evident that miRNAs play an intricate role in the modulation of autoimmune pathogenesis that involves itself being an effector and regulator simultaneously. A thorough discussion on miRNAs and epigenetics has recently been done by Sato et al. (2011) to further highlight the importance of miRNAs emerging from other studies.

## EPIGENETICS IN IMMUNE DISEASES

Immune diseases are typically described as disorders that cause hypo- or hyper- reactivity of the immune system. Autoimmune diseases fall into the category of immune system hyperactivity where the body produces autoantibodies and attack the “self,” which can cause extensive damage to various tissues, depending on the type of autoimmunity. The trigger to most autoimmune diseases in individuals is still unknown, but the growing research in monozygotic twins consistently shows that many diseases have relatively low concordance rate (<25%), indicating that genetics are not the only factor in inheriting and developing the disease. Similarly, genetic changes identified by GWAS so far account for only a small component of the heredity of autoimmune diseases. This has developed a set of evidence that suggests the environment and epigenetics play a crucial role in determining the development of these diseases in genetically susceptible persons.

Increasing evidence suggest a critical role of epigenetic determination in immune cell development and function. These include Th1/2 cell lineage development, regulatory T cell development and function, and the expression of multiple cytokines including IL-2 and TNFα.

### GENDER BIAS IN AUTOIMMUNE DISEASES

There is a clear gender bias in many autoimmune diseases (Whitacre et al., 1999; Ray and Yung, 2011; Ngo et al., 2014). For example, there are two to three times more women than men affected by RA and multiple sclerosis (MS). The ratio is even more acute in some other autoimmune diseases such as systemic lupus erythematosus (SLE) and Sjogren’s syndrome, with women outnumbering men by a ratio of 9–1. Endocrinological differences are important contributors to the sexual dimorphism seen in autoimmunity, including a role in gene-specific epigenetic modifications of DNA and histones (Kaminsky et al., 2006). DNA methylation is a central mechanism in silencing the majority of genes on one of the two X chromosomes in female cells (X-chromosome inactivation). Among females (XX) and the males who have Klinefelter’s syndrome (XXY), the frequency of SLE is ∼14 times higher in those having two copies of the X-chromosome than normal males (XY) who only possess a single copy of the X-chromosome. Evidence of defective DNA methylation or reactivation of silenced genes on the inactive X-chromosome in female lupus patients led to the theory that there exists a “gene-dose” effect from the X-chromosome which could also explain the similar susceptibility of SLE found in Klinefelter’s syndrome patients (Scofield et al., 2008). Similar to SLE, RA preferentially affects female patients who have skewed X-chromosome inactivation patterns. The same CD40L gene on the promoter of the X-chromosome was demethylated in female RA CD4+ T cells but was not observed in male RA patients (Liao et al., 2012). Over 80% of systemic sclerosis (SSc) patients are also female. Studies have shown that, in female SSc patients, the demethylation of elements that regulate CD40L on the inactive X-chromosome lead to overexpression of CD40L and may partially explain the gender bias (Lian et al., 2012).

## SYSTEMIC LUPUS ERYTHEMATOSUS

### DNA METHYLATION

Systemic lupus erythematosus is an autoimmune disease characterized by chronic or acute inflammation in multiple tissues of the body. The inflammation is in part due to production of pathogenic autoantibodies against nuclear self-antigens but the causes are still unclear. The first clue that epigenetic mechanisms may be involved in SLE pathogenesis came from the study of drug-induced lupus. The exposure to certain medications such as hydralazine and procainamide has been shown to induce SLE as early as the 1940s; this was one of the first pieces of observation that suggested environmental factors, acting through DNA methylation modifications, can affect SLE development (Richardson, 1986; Deng et al., 2003). Procainamide was subsequently shown to behave similarly as the DNMT1 inhibitor 5-azacytidine (5-azaC) to induce lupus-like disease in murine models (Yung et al., 1995, 1996). Hydralazine was shown to induce *in vivo* murine lupus via preventing DNMT1 upregulation by inhibition of the ERK (and potentially other) signaling pathways in T cells (Deng et al., 2003). Collectively, many studies on DNMTs have led to highlighting DNA methylation as an important epigenetic effect underlying SLE (Altorok and Sawalha, 2013).

Sunlight exposure is known to induce lupus activity, and photosensitivity remains a diagnostic criterion for the disease. A number of studies have examined the effects of UV (particularly UVB) irradiation on lupus and normal T cells, and showed that UV exposure can induce DNA hypomethylation and the upregulation of selected T cell genes implicated in lupus, including LFA-1 (CD11a) (Richardson et al., 1994; Zhu et al., 2013). An interesting study looked at the role of growth arrest and DNA damage-induced 45α gene (GADD45α) in lupus CD4+ T cells. Using ultraviolet B irradiation, they found that the T cells with increased expression of *gadd45A*, CD11a, and CD70 mRNA also had more autoreactivity and excessive B cell stimulation (Li et al., 2010). These results strongly support the theory that environmental triggers can cause the onset of autoimmune diseases; in this case, UV light was shown to be capable of inducing *gadd45A* which could then initiate a lupus flare. Next to this, it is the promotion of DNA demethylation in lupus CD4+ T cells that lead to the increased expression of *gadd45A*, showing that the environment and epigenetics work closely together to determine the expression of disease in an individual.

Some researchers have focused on identifying differential methylation patterns in T cells between lupus patients and healthy controls (Jeffries et al., 2011). Subsequently, hypomethylation of certain genes have been identified as important in lupus T cells, such as *ITGAL* (CD11a) (Lu et al., 2002), *CD40LG* (CD40L) (Callard et al., 1993), *TNFSF7* (CD70) (Oelke et al., 2004), Killer-cell immunoglobulin-like receptor (*KIR2DL4*) (Basu et al., 2009), perforin (*PRF1*) (Blanco et al., 2005), and *CD5* in lupus B cells (Garaud et al., 2009). Basu et al. (2009) showed that killer immunoglobulin like receptors (KIRs) that are normally expressed on natural killer (NK) cells are aberrantly expressed on demethylated CD4+ T cells similar to that observed in lupus CD4+ T cells. This suggests that the demethylation on KIR promoters caused a change in KIR expression that may be either stimulating or inhibiting the killing and secretion of inflammatory cytokines in SLE. A different study compared healthy B cells to lupus B cells and found that CD5-E1B, one of two CD5 isoforms, was demethylated in the presence of increased IL-6 stimulation—leading to the compromised expression of CD5-E1A and causing an expansion in the pool of autoreactive B cells (Garaud et al., 2009). Similarly, a separate study validated that the Erk/DNMT1 pathway is indeed defective due to the autocrine-loop that increases IL-6 signaling in lupus B cells; therefore, blocking this pathway allowed DNA methylation to occur and expression of HRES-1/p28 was restored (Fali et al., 2014). A number of genome-wide DNA methylation studies on CD4 T cells in lupus patients also recently been completed, and confirmed global DNA hypomethylation, and identified severe hypomethylation events near genes involved in type 1 interferon regulatory factor (IFN) signaling (Jeffries et al., 2011; Lin et al., 2012; Absher et al., 2013).

Interestingly, an earlier report suggests that treatment with 5-azaC, a DNA hypomethylation agent, may inhibit some features of autoimmunity in the MRL-*lpr* mouse model (Yoshida et al., 1990). However, the therapeutic effects appear to be specific to the one particular autoimmune mouse strain, suggesting that the protective effect may be specific to defects associated with the *lpr* gene.

### HISTONE MODIFICATIONS

Similarly, studies have delved into looking at histone methylation and acetylation statuses of lupus T cells. Using chromatin immunoprecipitation (ChIP), Zhang et al. (2010) looked at monocytes from controls and lupus patients to create gene expression arrays for H4 acetylation and found that it was significantly increased in lupus monocytes. Although the authors did not draw detailed conclusions on this observation, it was suggested that IFN-1 is in the affected pathway (Zhang et al., 2010). Hu et al. (2008) looked at the global histone H3/H4 acetylation and H3K4/H3K9 methylation in lupus CD4+ T cells and found global hypoacetylation in both H3 and H4 while global hypomethylation and H3K9 was detected. More convincingly, the degree of histone H3 acetylation correlated inversely with increased SLE disease activity (Hu et al., 2008; Patel and Richardson, 2010). Dai et al. (2007) had also identified that H3K27me3 marks play a role in the pathogenesis of SLE by looking at active and inactive SLE and RA patient peripheral blood mononuclear cells (PBMCs). Interestingly, MRL/lpr mice (spontaneously develop a lupus-like disease) that lack HDAC9 have decreased lymphoproliferation, less autoantibodies, and improved survival (Yan et al., 2011).

As histones are a major class of autoantigens in SLE (Burlingame et al., 1994), Liu et al. (2012) asked the question of whether post translational modifications (PTM) on histones in neutrophil extracellular traps (NETS) can induce autoantibodies that target these specific PTM of histones in SLE patients. Although they had no significant finding that can demonstrate this proposed mechanism between NETS and PTM of histones in SLE patients, they reported the presence of a significant autoantibody reactivity to acetyl-histone H2B (Liu et al., 2012).

### microRNA

A number of studies have examined the relationship between aberrant miRNA levels and lupus disease. One study found that the overexpression of miR-125 correlates to the reduction of DNMT1 protein expression in lupus CD4+ T cells (Zhao et al., 2011), and another study reported overexpression of miR-126 in healthy donor CD4+ T cells that led to hypomethylation and overexpression of genes CD11a and CD70 (Pan et al., 2010). Together, these studies demonstrate the importance of understanding the crosstalk between miRNA and DNA methylation as it regulates the activity of immune cells and affects disease progression. One study found that peripheral blood leukocytes from lupus patients express high levels of miR-21 and miR-198, and low expression of miR-184 and miR-17-5p (Dai et al., 2007). More recently, Lu et al. (2013) isolated T cells from five lupus patients and five healthy controls to compare the profiles of 270 human miRNAs, including miR-146a, miR-125a, miR-126, miR-21, and miR-148a, all miRNAs that have previously been associated with lupus pathogenesis (Tang et al., 2009; Pan et al., 2010; Zhao et al., 2010, 2011; Stagakis et al., 2011). Instead, they reported that there was a decrease expression of miR-145 and an increased expression of miR-224; consequentially, the drop in miR-145 expression in Jurkat cells led to the suppression of STAT-1 expression which is associated to lupus nephritis. On the other hand, the spike in miR-224 expression in Jurkat cells led to the decreased expression of the API5 molecule which is believed to facilitate cell apoptosis and contribute to lupus pathogenesis (Lu et al., 2013). Others have suggested that miR-146a may contribute to aberrant type 1 IFN expression in lupus by targeting signaling proteins (Tang et al., 2009). The number of reports linking different miRNA to autoimmunity is growing rapidly. At present, the precise pathogenic and therapeutic role of miRNA in lupus disease pathogenesis is unclear.

### EPIGENETIC THERAPEUTICS FOR SLE

Although much is now known about the epigenetic mechanisms of lupus and epigenetics-based therapies have entered into clinical practice, it is disappointing that much of the basic research in the field has not resulted in clinical trials in lupus or other autoimmune diseases.

A number of drugs used in clinical practice are known to inhibit HDACs, including valproic acid for epilepsy, and vorinostat and romidepsin for cutaneous T cell lymphoma. Although these have not been tried in lupus patients, it is interesting to note that there have been case reports of patients taking valproic acid developing lupus-like diseases. Although an American College of Rheumatology meeting abstract reported that valproic acid may prevent skin disease and reduce kidney disease severity in murine lupus, the results have not been formally published to date. Interestingly, others have reported that valproic acid may reduce proteinuria and the onset of glomerulosclerosis in a non-immune kidney injury murine models (Van Beneden et al., 2011; Brilli et al., 2013), suggesting that any beneficial effect of the drug may not be lupus-specific. HDAC inhibitors (HDACis) such as suberoylanilide hydroxamic acid (SAHA) and trichostatin A (TSA) have also been shown to ameliorate SLE disease (e.g., nephritis) in mice (Mishra et al., 2003; Reilly et al., 2008). TSA is largely an immunosuppressive imposer on T cells, but it is a promising potential in treating SLE as most research points to aberrant behaving T cells is caused by hypomethylation leading to overexpression of certain genes, fueling T cell activity.

An early study used cytarabine, a cytidine analog that also increases DNA methylation, as a treatment for refractory cutaneous lupus in a small pilot human study. Although there was relapse in the disease during the fourth week of 2 of the 3 tested cases, treatment with cytarabine was associated with the rapid improvement of refractory cutaneous lupus in all the treated patients (Yung and Richardson, 1995).

## RHEUMATOID ARTHRITIS

Rheumatoid arthritis is a complex autoimmune disease where synovial fibroblasts interact with immune cells that lead to joint destruction. The disease is characterized by synovial hyperplasia, production of selected autoantibodies such as rheumatoid factor and anti-citrullinated protein antibody, and systemic inflammation involving multiple organs outside the joints (McInnes and Schett, 2011; Choy, 2012). The RA synovial fibroblasts (RASFs) produce an array of inflammatory cytokines and chemokines which then further contributes and progresses the systemic inflammation. In a SCID mouse model, these synovial fibroblasts were capable of migrating from joint to joint, strongly suggesting that they are involved in encouraging local inflammation to spread systemically. More interestingly in the same study, it was proposed that these synovial fibroblasts carry an aggressive phenotype and can spread it to other joints when they “metastasize” and interact with the healthy synoviocytes (Lefevre et al., 2009).

### DNA METHYLATION

The earliest clue suggesting an etiologic role of DNA methylation in RA came from a 1990 study showing T cells from patients with RA have hypomethylated DNA (Richardson et al., 1990; Sanchez-Pernaute et al., 2008). A subsequent study found retrotransposon elements in rheumatoid synovial fluid that are normally regulated by DNA methylation (Neidhart et al., 2000). Others have since reported hypomethylated IL-6 promoter in RA peripheral blood cells (Nile et al., 2008). A recent genome-wide methylation study of peripheral blood cells from RA patients also implicated aberrant DNA methylation as a key pathogenic process in the disease (Liu et al., 2013).

Both DNA hypomethylation and hypermethylation have been reported in RASFs, and its activation and aggressive phenotypes are associated to epigenetic differences between the healthy control, RA patients, and OA patients. The synovial fibroblasts were found to have different DNA methylome signatures when comparing RASFs and OASFs to healthy synovial fibroblasts; however, between RASF and OASF, their methylome signatures were also distinguishable from one another. The unique aggressive phenotype attributed to RASF may be a useful tool for diagnosing patients and possibly become a therapeutic target. More importantly, among the 207 identified hypo- or hyper- methylated genes in fibroblast-like synoviocytes isolated from the site of RA disease patients, hypomethylation was traced to multiple pathways that relate to cell migration, focal and cell adhesion, transendothelial migration, as well as extracellular matrix interactions (Nakano et al., 2013). Any methylation changes at these genes can therefore potentially affect RA disease progression. Another study found that a significant reduction in *S*-adenosyl methionine (SAM) in RASFs as compared to OASFs may be due to increased recycling of polyamines and explain the global DNA hypomethylation observed in RASFs (Karouzakis et al., 2012).

### HISTONE MODIFICATIONS

Trenkmann et al. (2011) obtained OASFs and RASFs to study the expression, regulation, and function of the histone methyltransferase enhancer of zeste homolog 2 (EZH2). EZH2 was overexpressed in RASFs as compared to OASFs, and in pursuit of the downstream effectors of this pathway, they found that secreted frizzled-related protein 1 (SFRP1), an inhibitor of Wnt signaling that is associated with the activation of RASF was its target gene. And although EZH2 is capable of generating the trimethyl mark on H3K27, its overexpression did not seem to influence global histone trimethylation in the RASFs (Trenkmann et al., 2011; Klein and Gay, 2013).

Grabiec et al. (2010) set out to investigate whether the administration of HDACis would counterproductively induce further inflammation in diseases such as asthma, chronic obstructive pulmonary disease, or RA. They used TSA and nicotinamide, both HDACis, to inhibit class I/II HDACs or class III sirtuin HDACs and found that they could block the production of IL-6 and TNF-a in macrophages from both RA patients and healthy controls. This finding supports the notion that HDACis can selectively and effectively suppress proinflammatory growth factors, chemokines and cytokines that fuel RA patient disease progression (Grabiec et al., 2010).

### microRNAs

Stanczyk et al. (2008) found that the increased expression of miR-155 and miR-146a in RASFs treated with TNFα was higher than those in OASFs. From these findings, the authors conclude it is possible that the inflammatory environment can be altering miRNA expression profiles in resident cells of RA joints, spreading the phenotype. In a different study with the same lab, they reported elevated expression of miR-203 in RASFs than in OASFs and healthy controls. Furthermore, they found that DNA demethylation achieved via 5-azaC also elevated the expression of miR-203, showing that there is methylation-dependent regulation of miR-203 expression in RASFs (Stanczyk et al., 2011). More recently, Murata et al. (2013) reported that they found higher plasma concentrations of miR-24 and miR-125a-5p in RA patients than in either SLE or OA patients; therefore, they are also potential diagnostic markers of RA.

An interesting study set out to define miRNAs expression profiles of naïve and memory regulatory T cells (Tregs), including conventional naïve and memory T cells (Tconvs) in RA patients and healthy controls. They reported finding unique miRNA expression signatures that could provide a subclassification of T-cell subsets associated to the disease. Specifically, they were able to identify 5 miRNAs, miR-146a, miR-3162, miR-1202, miR-1246, and miR-4281, to be significantly enriched in both naïve and memory Tregs. On the contrary, three miRNAs, miR-142-5p, let-7c, and miR-590-5p were menially present in both Treg subsets (Smigielska-Czepiel et al., 2014). This study demonstrated that the inflammatory state of RA joints may be influenced by the T cell subset composition in the synovial fluid.

### EPIGENETIC THERAPEUTICS FOR RHEUMATOID ARTHRITIS

There have been many experimentally used drugs that target epigenetic mechanisms that contribute to RA pathogenesis. DNMT inhibitors such as 5-azaC was used to treat normal SF and this reproduced RASF phenotype (Kooistra and Helin, 2012). TSA and nicotinamide, both HDAC inhibitors, were separately used to reduce TNF-α which in turn reduces IL-6 expression in macrophages isolated from the PBMCs of RA patients (Gillespie et al., 2012). Others have shown that targeting HDACs, using HDACi such as valproic acid, can mitigate the severity of murine chronic inflammatory arthritis (Chung et al., 2003), in part via improving regulatory T cell function (Saouaf et al., 2009). These show potential therapeutic value, because they can reduce or disrupt the production of inflammatory signals that fuel RA disease activity. Another promising study silenced SIRT1 and promoted apoptosis in RASFs from synovial tissues and cells from RA patients (Niederer et al., 2011).

## SYSTEMIC SCLEROSIS

Systemic sclerosis is a rare autoimmune disease with an incidence of 0.002% but severe in its pathology—the 9 year survival rate when internal organs are involved is 39% (Nikpour et al., 2010). It is characterized by microvascular dysfunction, progressive fibrosis of skin and/or internal organs, and immune abnormalities in microvascular endothelial cells, T lymphocytes, B lymphocytes, and fibroblasts. Although the disease’s cause is still widely unknown, microvascular injury is believed to begin the cascade of events in disease progression. As the microvascular injury leads to endothelial activation, leukocytes are recruited and leads to T and B lymphocytes to secrete a cocktail of cytokines, chemokines, and autoantibodies. These agents then activate fibroblasts for wound healing which may then lead to tissue fibrosis (Varga, 2008; Castro and Jimenez, 2010).

### DNA METHYLATION

Abnormalities in DNA methylation is associated with SSc. DNMT inhibitors such as azacytidine can demethylate endothelial nitric oxide synthases (eNOS) which is reduced in SSc patients, and thus, is a possible therapeutic target (Matouk and Marsden, 2008). Other studies look at DNA methylation in fibroblasts that contribute to the excessive deposition of collagen and extracellular matrix and how DNMT inhibitors such as 5-aza-2′-deoxycytidine (2-deoxy-5-azaC; 5-Aza-CdR; decitabine) can reverse this pathology in SSc fibroblasts (Wang et al., 2006). More recently, a study isolated dermal fibroblasts in six diffuse cutaneous SSc (dSSc) patients, six limited cutaneous SSc (ISSc) patients and compared them to healthy controls and identified thousands of differentially methylated CpG sites in both dSSc and ISSc patients. They performed pathway analysis of the shared differentially methylated genes and reported a significant upregulation in a set of genes involved with extracellular matrix-receptor interaction and focal adhesion (Altorok et al., 2014b). Altogether, the data provides a useful platform for future studies to look at novel therapeutic targets for both forms of the disease.

### HISTONE MODIFICATIONS

A study that started out trying to identify autoantibodies in the sera of SSc patients instead revealed the lack of autoantibody against HDAC-3 which is detected in normal individuals. They concluded that the anti-HDAC-3 antibody must be protective in nature (Kuwatsuka et al., 2009). Another group investigated histone modifications in B cells to study their potential role in SSc pathogenesis, and they found global histone H4 hyperacetylation and global histone H3K9 hypomethylation. These changes in histone modifications were correlated to higher expression of JHDM2A and downregulation of HDAC2, HDAC7, and SUV39H2. Importantly, global H4 acetylation positively correlated with disease activity while HDAC2 was negatively correlated to skin thickness (Wang et al., 2013). This was a promising finding, as an epigenetic machine was indirectly found to control a symptom found in SSc pathogenesis.

### microRNAs

Another approach researchers have taken is to look at miRNAs that can influence the development of SSc fibrosis. miR-21, miR-31, miR-146, miR-503, miR-145, and miR-29b have been linked to SSc fibrosis. miR-21 and miR-145 have already been identified as regulators of genes related to fibrosis such as, *SMAD7*, *SAMD3*, and *COL1A1* (Zhu et al., 2012a). Honda et al. (2012) reported that in SSc fibroblasts, miR-196a expression is TGF-b mediated, and miR-196a inhibitor leads to the overexpression of type I collagen in normal fibroblasts. This correlated with how patients who had lower serum miR-196a levels had a much more exacerbated set of SSc symptoms than those who did not have lower miR-196a levels (Honda et al., 2012). Makino et al. (2012) analyzed serum samples from SSc, SLE, and several other diseases to compare to healthy controls in order to identify useful diagnostic biomarkers; miR-142-3p serum levels were significantly higher in SSc than all others, making it a potential SSc biomarker.

### EPIGENETIC THERAPEUTICS FOR SYSTEMIC SCLEROSIS

The data summarized above support a scenario where aberrant epigenetic control may lead to the excessive accumulation of dermal extracellular matrix proteins that is the hallmark of SSc. Wang et al. (2006) demonstrated that HDAC inhibitors can repress collagen suppressor gene FL11 and restore normal collage expression in SSc fibroblasts. The promoter of FL11 is hypermethylated in SSc fibroblasts which leads to suppression of its transcription; therefore incubating it with 5-aza increases its transcription and decreases release of collagen (Jungel et al., 2011). This is solid evidence that portrays the anti-fibrotic effect of DNMT inhibitors. TSA primarily reduces all transcripts of HDACs and is especially potent toward the inhibition of HDAC7 (Dokmanovic et al., 2007), possibly making it a specific and targetable component of SSc pathogenesis.

## SJÖGREN’S SYNDROME

Sjögren’s syndrome (SS; SjS) is an autoimmune disease characterized by the dysfunction of exocrine glands and lymphocytic infiltrations, particularly the labial (minor salivary) and lacrimal glands which leads to the hallmark symptoms of SS—xerostomia (dry mouth) and keratoconjunctivitis sicca (dry eyes). Other clinical manifestations involve parenchymal organs such as the kidney, lung, and liver (Mavragani and Moutsopoulos, 2014). Primary SS (pSS), where the disease occurs alone, can be characterized via the detection of circulating autoantibodies against the sicca syndrome (SS) and ribonucleoprotein (RNP) particles (Ro/SSA and La/SSB). On the other hand, secondary SS (sSS) is when the disease is accompanied by other autoimmune diseases such as RA or SLE and finding biomarkers to distinguish between patients who have sSS remains a challenge.

### DNA METHYLATION

Similar to a subset of autoimmune diseases, demethylation agents have long been demonstrated as potent inducers of SS. Cannat and Seligmann (1968) administered hydralazine and isoniazid to mice, and this resulted in the development of SS accompanied by SLE and RA immunological features that simultaneously exhibited “antinuclear factors (ANF).” These antinuclear antibodies disappeared in certain mice 2 months after the discontinuation of drug treatment. Since then, several groups have looked at the global DNA methylation in several types of SS cells. Thabet et al. (2013) recently showed that there is a reduced global DNA methylation in salivary gland epithelial cells (SGEC), peripheral T cells, and B cells from pSS patients when compared to healthy control biopsies. This reduction was associated to a sevenfold decrease in DNMT1 as well as a twofold increase in Gadd45-α, strongly indicative of a pathogenic role for epigenetics in SS. In the same study, an *in vitro* co-culture experiment showed that DNA hypomethylation in SGECs may be in part due to B cells causing alterations to the PKCδ/Erk/DNMT1 pathway. Therefore, the authors concluded that infiltrating B cells may be contributing to the demethylation process—congruent to the findings from an older study that showed that SS patients treated with rituximab (an anti-CD20 antibody that depletes B cells) led to higher global DNA methylation levels after 4 months of drug administration (Devauchelle-Pensec et al., 2007).

Two separate studies reported that treatment with 2-deoxy-5-azaC increases aquaporin 5 (AQP5) expression in the human salivary gland (SG) ductal cell line NS-SV-DC (Motegi et al., 2005) and restores salivary function in the murine aging model C57Bl/6CrSlc (Yamamura et al., 2012). However, contrary to the notion of using demethylating drugs to restore salivary function, a more current study confirmed that AQP5 is not repressed but overexpressed in SGEC of SS patients, and it was shown that anti-muscarinic type 3 receptor (α-M3R) suppresses AQP5 trafficking to the membrane which contributes to the keratoconjunctivitis sicca in SS patients (Lee et al., 2013). Altorok et al. (2014a) performed a genome-wide DNA methylation analysis of naïve CD4+ T cells from pSS patients and reported 553 hypomethylated and 200 hypermethylated CpG sites when compared to healthy controls. The highlighted differentially methylated genes included *LTA* (encodes for lymphotoxin α or TNF-β), *RUNX1* (runt-related transcription factor that may be linked to lymphoma predisposition in SS patients), a subset of interferon signature pathway genes, and also a group of genes that encode for membrane water channel proteins. Yin et al. (2010) had shown that similar to SLE patients, CD70 (*TNFSF7*) in pSS CD4+ T cells are hypomethylated and thus overexpressed, contributing to autoreactivity.

### HISTONE MODIFICATIONS

Although the effect of CD70 upregulation in pSS CD4+ T cells has yet to be investigated, Zhou et al. (2011) analyzed the histone modifications in the *TNFSF7* promoter region of SLE patients and linked it to a global histone H3 and H4 hyperacetylation and increased dimethylation of H3 lysine 4 (H3K4me2). Both these results positively correlated to disease activity. Because SS is closely associated to SLE, findings like these and all the other chromatin links to SLE disease, suggests that targeting aberrant histone modifications in SS patients may also be a feasible treatment pathway (Konsta et al., 2014).

### microRNAs

Similarly to histone modifications, very little research has been performed to study the miRNA regulation of SS progression, their potential role in disease, and as targets of therapy. It is however gaining attention. For example, Pauley’s group linked increased expression of miR-146a in human monocytic THP-1 cells to an increase in phagocytic activity and suppression of inflammatory cytokine production, and this finding was used to deduce that the increase in miR-146a expression in SS patients may be contributing to the pathogenesis of the disease (Pauley et al., 2011). The expression of miR146a and miR146b were measured along with their downstream target genes IRAK1, IRAK4, and TRAF6 in PBMCs of pSS patients to compare to healthy controls. The study reported overexpression of miR146a/b and TRAF6 while IRAK1 expression was decreased, leading them to propose TRAF6 as a more specific SS biomarker than miR146a/b (Zilahi et al., 2012). Other groups have been focusing on identifying that there are differentially expressed miRNAs between the minor SGs of SS patients and those of healthy controls (Alevizos et al., 2011; Tandon et al., 2012), and these studies have established that miRNAs are indeed potential biomarkers of SS. More interestingly, Alevizos et al. (2011) used Ingenuity Pathways Analysis to predict the most likely targets of some of these miRNAs and found that various neurologic function were the main targets—leading the authors to suggest that the miRNA control over neurologic regulation of SGs may be the underlying pathogenesis of SS. Among SGs, SGECs, and PBMCs of SS patients and controls, Kapsogeorgou et al. (2011) reported that certain miRNAs (prediction analysis resulted in 11 human miRNAs that were likely to target Ro/SSA and La/SSB autoantigens) were differentially expressed. The authors pointed out that the elevated expression of miR-181a, miR-200b, and miR-223 in SS may be part of a negative feedback loop that regulates autoantigen expression of Ro/SSA and La/SSB.

## TYPE 1 DIABETES

### DNA METHYLATION

Type 1 diabetes (T1D) is an autoimmune disease where T-cell mediated destruction of insulin-secreting β cells causes insulin deficiency and leads to an increased level of glucose in blood and urine. The disease concordance rate in monozygotic twins ranges between 13 and 67.7%, and this has been taken to suggest that both environmental factors and epigenetics are potentially involved (Huber et al., 2008). However, the large range of variation in risk may also be due to human leukocyte antigen genotypes, and studies show that low-risk HLA genotypes do correlate to less destruction of the β cells (Spoletini et al., 2007). Nonetheless, researchers have found differences when investigating the differential methylation patterns between T1D patients and healthy controls in the seven CpGs proximal to the insulin gene promoter (Fradin et al., 2012). Another recent study has shown that T1D patients have decreased DNA methylation in the insulin-like growth factor binding protein-1 (IGFBP-1) that correlates with increased circulating IGFBP-1 levels and the presence of diabetic nephropathy (Gu et al., 2014).

### HISTONE MODIFICATIONS

Aside from DNA methylation, T1D pathology has also been associated to histone modifications. A study that used curcumin to observe changes in histone modification in diabetic rats were fruitful and found that next to increasing histone acetylation on H3, it significantly decreased blood urea nitrogen, creatinine, and increased albumin—all of which are markers of diabetic nephropathy in T1D (Tikoo et al., 2008). Miao et al. (2014) used ChIP to profile H3K9 acetylation, H3K4 trimethylation, and H3K9 dimethylation in blood monocytes and lymphocytes from diabetes patients. The aim was to see if epigenetics is associated to glycemic history and metabolic memory in T1D patients, and the results allowed the authors to claim that there is at least potential in linking epigenetic changes to metabolic memory in T1D (Miao et al., 2014).

### microRNAs

Various miRNAs have been linked to the pathogenesis of T1D and are associated to β-cell death (Dang et al., 2013). This has been strongly supported by studies such as the elucidation on the NF-κB-miR-21-PDCD4 axis that regulates pancreatic β cell death. The presence of miR-21 decreases the level of PDCD4 which can enable cell death, and there can essentially be a “block” to the destruction of the precious β cells that secrete insulin (Ruan et al., 2011). The growing interest in linking miRNA regulation to T1D pathogenesis has led a group to analyze Tregs and other T cells in diabetic patients, and it was found that miRNA-146a had enhanced expression while eight other miRNAs, miR-20b, miR-31, miR-99a, miR-100, miR-125b, miR-151, miR-335, and miR-365 had decreased expression in Tregs (Hezova et al., 2010). Developing this type of miRNA signature in Tregs is important for expanding the possibility of using miRNAs as therapeutic targets in T1D.

### EPIGENETIC THERAPEUTICS FOR T1D

While there have been little research done on testing epigenetic drugs on T1D models, there was a study that inhibited class I and class II HDACs with TSA which led to an enrichment of endocrine progenitor cells and β cells. On the contrary, inhibiting class I HDACs with valproic acid instead enhances endocrine progenitor and α-cell pool (Haumaitre et al., 2008; Bramswig and Kaestner, 2012). As Bramswig concisely placed it, further work must be done so that diabetes-specific epigenetic profiles will be clearly established to distinguish between non-, early-, and late- diabetic states in order to develop more specific and effective markers for targeting.

## MULTIPLE SCLEROSIS

### DNA METHYLATION

Multiple sclerosis is characterized as a chronic inflammatory, neurodegenerative disease of the brain and spinal cord. Its cause is largely unknown, although there are genetic inheritance implications, the low concordance rate in identical twins of 6–30% have led many to believe that there are other etiological factors and epigenetics at play (Koch et al., 2013). Epigenetics research in MS has primarily focused on miRNA, but there are studies that have begun to point at the significance of DNA methylation in MS as well. A study analyzed 56 genes for differential methylation between healthy controls and patients with MS and found that 15 of the 56 genes contained methylation statuses that could be used to distinguish between MS patients who are in remission and those in later, more exacerbated stages of the disease (Liggett et al., 2010). Other promising research looking at the role of epigenetics in MS have identified the overexpression of DNMT3a as a possible cause of neuronal cell death due to its ability to induce apoptosis in those cells. Although it has not yet been implemented in the EAE or any other models of MS, the authors interestingly pointed out that DNMTs, enzymes that participate in the mechanism that regulate DNA methylation in neurons, could be equivalently seen as a mechanism of neurodegeneration (Chestnut et al., 2011).

### HISTONE MODIFICATIONS

Similar to Liggett’s study, Pedre et al. (2011) looked at histone acetylation patterns in the white matter and early MS lesions to look for therapeutic targets. The study reported that chronic MS patients shifted toward acetylation, but this especially occurred in a subset of female patients. Most intriguing was that a significant level of acetylation of H3 was detected in the peripheral white matter (PPWM) but decreased H3 acetylation was reported in remyelinating lesions. This finding could lead to biomarkers that distinguish between different types or stages of remyelination, as the authors had pointed out.

### microRNAs

A study developed miRNA expression profiles from patient whole-blood samples that could help differentiate patients in the relapsing-remitting (RMMS) phase of the disease from healthy individuals with the use of miR-145 as a biomarker of RMMS (Keller et al., 2009). Researchers looking at miRNA for potential epigenetic therapeutics have observed that miRNA dysregulation seems to favor proinflammatory activity which promotes disease progression. The miRNA profile of active and inactive MS lesions were analyzed and found to contain 28 miRNAs in active lesions and 35 miRNAs in inactive lesions that were dysregulated when compared to healthy controls (Junker et al., 2009). miRNA expression profiling have inspired many other studies to attempt narrowing down the pathological effects of miRNA dysregulation. It has been shown that miR-155 and miR-326 are associated with T-cell differentiation, and this led to a study that studied how miR-155 enhances inflammatory T cell development in autoimmune diseases—reporting high levels of miR-155 in activated CD4+ T cells that could also possibly contribute to the production of inflammatory cytokines in dendritic cells (O’Connell et al., 2010).

### EPIGENETIC THERAPEUTICS FOR MULTIPLE SCLEROSIS

Histone deacetylases inhibitor, such as TSA, has reduced spinal cord inflammation, demyelination, neuronal and axonal loss in EAE models, and thus it has potential as a treatment option for MS (Camelo et al., 2005). However, HDAC inhibitors used at the systemic level have shown to negatively affect the generation of new myelin, because HDAC is crucial to developmental myelination (Marin-Husstege et al., 2002; Shen et al., 2005).

Chirivi et al. (2013) have proposed citrullination as a target for epigenetic intervention in MS and other inflammatory diseases; they postulate that shielding citrullinated histone epitopes, which play a large role in NET formation, can help prevent the intensification of the inflammatory response in MS and other inflammatory diseases.

## OBESITY AND TYPE 2 DIABETES

Obesity is closely associated with the development of both type 2 diabetes (T2D) and metabolic syndrome. There is growing evidence that other than individual lifestyle choices, developing obesity is also in part due to genetic disposition, especially epigenetic processes. The “thrifty phenotype hypothesis” proposes that the association between low birth weight and an increase in risk of later disease is due to the fetus making adaptations (e.g., insulin resistance) to survive the maternal malnourishment, leading to a phenotype that would have to cope with a different, more aﬄuent postnatal environment (Hales and Barker, 1992). Increased DNA methylation has been associated with T2D in a Swedish population (Gu et al., 2013). Interestingly, global DNA hypermethylation has been linked to the presence of diabetic retinopathy in T2D (Maghbooli et al., 2014). BCL11A gene polymorphism has been established as a risk for T2D, and a recent study suggests a possible male gender-specific association between BCL11A gene methylation and T2D (Tang et al., 2014). Additionally, factors such as cholesterol and the different types of fatty acids in the diet can also impact on genome-wide DNA methylation patterns (Voisin et al., 2014).

Two developmental scenarios are possible: either nutritional limitation or excessive nutrition during early life, such as embryo or fetal development, can play a role or increase their risk in certain diseases (Gluckman and Hanson, 2008). In order to exploit this mechanism, studies have challenged fetal development in various animal models, such as rodents by reducing specific maternal nutrition intake or administering a high fat diet to observe the offspring phenotypic outcomes. Another study looked at how the outcome of a low-nutrition prenatal diet paired with post-weaning high fat diet influenced adiposity in the offspring; the result was that there was significantly higher adiposity in the combination conditions, as compared to the high fat diet alone (Vickers et al., 2000). Recently, a GWAS on 459 individuals of European origin was performed to explore the relationship between DNA methylation and BMI, and the analysis highlighted that increased BMI in these individuals is linked to an increase in methylation at the *HIF3A* locus in blood cells and in adipose tissue (Dick et al., 2014). This study generates a lot of support for the relationship between the epigenome and the development of obesity in genetically susceptible individuals.

## PRENATAL NUTRITION

During normal development, DNA methylation is crucial for silencing specific genes at different stages and in differentiating tissues. As such, maternal intake of macronutrients and micronutrients are believed to play a crucial role in the infant’s development and also its adult susceptibility to late-life diseases.

Much of the current research has focused on the long term effects of gestational macronutrition (calorie, fat, and protein) in the offspring. Most human and animal studies have found that almost any maternal nutrition stressors during pregnancy increase the offspring’s risk of obesity, T2D, insulin resistance, high blood pressure and heart disease. Additionally, paternal diet may also play a role in determining the health of the offspring. An interesting study showed that paternal high-fat diet can cause pancreatic beta cell dysfunction in female offspring, likely through epigenetic mechanisms (Ng et al., 2010). Interestingly, children born during the Dutch Hunger Winter in Second World War have different DNA methylation patterns, including hypomethylation of the IGF2 gene, compared to their siblings born with better gestational nutrition (Heijmans et al., 2008). Another study also found gender-dependent increased DNA methylation of IL-10, LEP, ABCA1, GNASAS, and MEG3 genes in these subjects exposed to prenatal famine (Tobi et al., 2009).

One of the hallmarks of the effect that maternal dietary supplements can have on the offspring is vested in the viable yellow agouti (*A^vy^*) mouse model. The Agouti gene encodes a paracrine signaling molecule that produces either black eumelanin (a) or yellow pheomelanin (A). The *A^vy^* metastable epiallele resulted from the insertion of an intracisternal A particle (IAP) murine retrotransposon upstream of the transcription start site of the Agouti gene, which in turn serves as an epigenetic biosensor for nutritional and environmental alterations on the fetal epigenome. Wolff et al. (1998) observed that upon supplementing the agouti mice excess folic acid, vitamin B12, choline, and betaine prior to pregnancy and then throughout, the offspring was veered away from the phenotype of obesity, diabetes, and susceptibility to tumors due to differences in methylation on the IAP retrotransposon.

It is believed that maternal prenatal diets enact long-term consequences through epigenetic mechanisms that can aid in the offspring’s protection against diseases. Dietary factors can directly alter the source or availability of methyl donors which can then influence the developmental process. DNA methylation depends on the levels of folic acid, methionine, choline, betaine, vitamins B2, B6, and B12, and therefore, any dietary imbalance of these agents can cause a change in methylation patterns (Haley et al., 2011).

Delaney et al. (2013) showed recently that maternal micronutrient supplementation with methyl-donors can protect F1 ApoE -/- mice against atherosclerosis by inhibition of T cell Ccr2 expression, a critical chemokine receptor that is central to the pathogenesis of atherosclerosis. However, the study went on to demonstrate that prolonged exposure to high-fat diet can override the protective phenotype from the maternal prenatal diet (Delaney et al., 2013). In contrast, a similar prenatal diet was found to increase the severity of allergic airways disease in a mouse model of asthma that persisted into the F2 generation (Hollingsworth et al., 2008), and another study showed enhanced colonic inflammation in a mouse model of inflammatory bowel disease (Schaible et al., 2011). Besides the effect of the micronutrient diet on DNA methylation, it is possible that the diet may exert its effect via other epigenetic mechanisms or through changes in the gut microbiome.

Intestinal commensal microbes have been key regulators of immune system development and homeostasis (Ivanov and Honda, 2012). The simplest demonstration of this was shown in germ-free (GF) mice that struggle to develop gut-associated lymphoid tissues (GALTs) and isolated lymphoid follicles (Hamada et al., 2002; Bouskra et al., 2008), indicating the necessity of the presence of intestinal commensals for proper development of immune components within the gut. Through subsequent studies, different commensals have been identified as crucial to the induction and regulation of various immune cell populations such as regulatory T cells (Tregs) (Feehley et al., 2012; Arpaia et al., 2013; Furusawa et al., 2013), Th17 effector T cells (Ivanov et al., 2008, 2009; Gaboriau-Routhiau et al., 2009; Yang et al., 2014), and a more exhaustive list has been assembled by Kamada and Nunez (2014). Markle et al. (2013) showed in a non-obese diabetic NOD mouse model of type 1 diabetes that the transfer of gut microbiota from adult males to immature females led to the alteration of recipient microbiota. Following this, testosterone and metabolomic changes were associated to T1D protection in the female pups through the decrease in insulitis and insulin autoantibodies. This study emphasized the ability of the commensal microbiobes to alter sex hormone levels which in turn influenced autoimmune disease susceptibility in the context of gender and its gene-associated risks.

## ESTROGEN, EPIGENETICS, AND AUTOIMMUNE DISEASES

The gender bias seen in many autoimmune diseases has pointed to a role for sex hormones in the pathogenesis of these diseases. Much have already been written about the role of estrogens and androgens in promoting autoimmune diseases through direct effects on proinflammatory cytokine production. More recently, it has been shown that epigenetic-mediated estrogen signaling also plays a role in the human development (Jiang et al., 2013) and in the pathogenesis of gender-dependent diseases (Frick et al., 2011; Mann et al., 2011; Hervouet et al., 2013). Interestingly, bisphenol A (BPA), an environmental estrogenic compound, is well known to affect DNA methylation, disrupt imprinting (Susiarjo et al., 2013), and worsen lupus disease in animal models (Sawai et al., 2003).

## SUMMARY AND CONCLUSION

Over the past two decades, research has been vested in identifying epigenetic therapeutic targets in cancer and designing epigenetic therapies to tackle oncological pathways, but growing evidence showing that epigenetic pathways are pertinent to many other non-oncological diseases have drawn the interest of scientists (**Table 1**). The age of cancer research has observed a number of UFDA-approved drugs, including epigenetic drugs such as HDAC inhibitors that were meant to maintain or normalize acetylation states in cancer cells that have aberrant acetylation patterns. Aforementioned, later studies have demonstrated that HDAC inhibitors regulate a vast number of proteins of which only a small percentage consists of histone proteins. This realization opens both a potential to use HDAC inhibitors for other diseases, like lupus and RA where it has been shown to reduce proinflammatory cytokines, and it also raises awareness in the potential backlash of targeting such a versatile and broad epigenetic regulator.

**Table 1:** Targets of epigenetic modifications associated with non-cancerous conditions.

	Cell type	DNA methylation	Histone modifications	microRNA	Epigenetic therapies
Systemic lupus erythematosus (SLE)	CD4+ T cells, B cells (CD5-F1B), natural killer cells, monocytes	UVB induces DNA hypomethylation, GADD45α, CD11a, CD70, CD40LG, TNFSF7, KIR2DL4, PRF1	IFN-1, H3/H4 acetylation on H3K4/H3K9 (global hypomethylation on H3K9)	miR-125, miR-126, miR-21, miR-198, miR-184, miR17-5p, miR-146a, miR-125a, miR-126, miR-21, miR-148a, miR-145 (Jurkat), miR-224	HDAC inhibitors (SAHA, TSA), cytarabine
Rheumatoid arthritis (RA)	T cells, RA synovial fibroblasts (RASFs)	IL-6 promoter, reduction in *S*-adenosyl methionine (SAM) pool leads to hypomethylation	EZH2, SFRP1 (Wnt signaling) which affects H3K27 trimethylation	miR-155, miR-146a, miR203, miR-24, miR-125a-5p, miR-3162, miR-1202, miR-1246, miR-4281, miR-142-5p, let-7c, miR-590-5p	HDAC inhibitors (TSA, nicotinamide)
Systemic sclerosis (SSc)	T and B lymphocytes, fibroblasts	DNMT inhibitors (azacytidine) can demethylate eNOS	HDAC-3, HDAC2, HDAC7, SUV39H2, global H4 acetylation	miR-21, miR-31, miR-146, miR-503, miR-145, miR-29b, miR-196a, miR-142-3p	DNMT inhibitors (2-deoxy-5-azaC)
Type 1 diabetes (T1D)	T cells, pancreatic β cells	Insulin gene promoter, IGFBP-1	H3 acetylation, H3K4 trimethylation, H3K9 dimethylation	miR-fb-mIR-PDCD4 axis, miR-20b, miR-31, miR-99a, miR-100, miR-125b, miR-151, miR-335, miR-365	HDAC inhibitors (TSA), valproic acid
Multiple sclerosis (MS)	Neuronal cells, peripheral white matter (PPWM), remyelinating lesions, T cell differentiation	Overexpression of DNMT3a associated with neuronal cell death	Acetylation occurs in a subset of female patients. H3 acetylation in PPWM but reduced in remyelinating lesions	miR-145 (RMMS marker), miR-155, miR-326 (both in T cells)	HDAC inhibitors (TSA), citrullination and NETs as a possible target
Obesity and type 2 diabetes (T2D)	Adipose tissue, blood cells	Global DNA hypermethylation (diabetic retinopathy), BCL11A (male specific association), HIF3A locus methylation	–	–	Prenatal diets (folic acid, methionine, choline, betaine, vitamins B2, B6, B12)

As many others have mentioned, the difficulty in utilizing epigenetics in therapy is that these epigenetic regulators affect more than the target of interest and has dose-limiting toxicities (Gray and De Meyts, 2005). Next to this obvious problem, there is a constant struggle to hunt for high-specificity biomarkers and to also design therapeutics that are capable of targeting these specific markers. In many instances, while individual illnesses have been associated with epigenetic changes it is not always clear whether these associations are the cause or the consequence of the diseases. Despite these complications, many research studies that were highlighted in this review have brought exciting and promising evidence that epigenetics are applicable beyond cancer therapy. GWAS are now progressing with the inclusion of the epigenome to establish even more powerful sets of data that can help distinguish non-Mendelian inheritance patterns. As Best and Carey (2010) pointed out, epigenetic therapies carry the potential to treat the disease as opposed to treating the symptoms of the disease—to which many current therapies are limited.

Understanding the epigenetic basis of disease may also be important to disease management besides identifying epigenetic changes as potential therapeutic targets. It is understood that most complex diseases develop as a result of multiple cumulative genetic factors interacting with beneficial or harmful environmental agents. It is conceivable that, in the new era of personalized medicine, epigenetic research will lead to the development of individualized epigenomic profiling that can both inform risk (e.g., risk of lupus or RA in a first degree relative of an affected individual) and guide therapies (e.g., which anti-inflammatory or biologic agents, or likelihood of developing drug side-effects), much like that is being promised by personalized genomic-based medicine.

## Conflict of Interest Statement

The authors declare that the research was conducted in the absence of any commercial or financial relationships that could be construed as a potential conflict of interest.
